# The Short-Term Load Forecasting for Special Days Based on Bagged Regression Trees in Qingdao, China

**DOI:** 10.1155/2021/3693294

**Published:** 2021-09-15

**Authors:** Huanhe Dong, Ya Gao, Yong Fang, Mingshuo Liu, Yuan Kong

**Affiliations:** College of Mathematics and Systems Science, Shandong University of Science and Technology, Qingdao 266590, China

## Abstract

There are many factors that affect short-term load forecasting performance, such as weather and holidays. However, most of the existing load forecasting models lack more detailed considerations for some special days. In this paper, the applicability of the bagged regression trees (BRT) model combined with eight variables is investigated to forecast short-term load in Qingdao. The comparative experiments show that the accuracy and speed of forecasting have some improvements using the BRT than the artificial neural network (ANN). Then, an indicator variable is newly proposed to capture the abnormal information during special days, which include national statutory holidays, bridging days, and proximity days. The BRT model combined with this indicator variable is tested on the load series measured in 2018. Experiments demonstrate that the improved model generates more accurate predictive results than BRT model combined with previously variables on special days.

## 1. Introduction

Accurate short-term forecasts of electricity load are essential for the real-time scheduling of power systems, optimizing operational costs, and improving the reliability of distribution networks. Specially, these forecasts have been playing a crucial role in unit commitment and maintenance, power interchange, and task scheduling of both the power generation and distribution facilities. Economically, the high precision in load forecasts can allow utilities to operate at minimum cost, which may contribute to significant savings in electric companies. Therefore, improving the accurate level of short-term load forecasting could not only increase the management efficiency in terms of schedule planning but also reduce the energy budgets, which is an encouraged behavior for those resource-saving developing countries [1, 2].

Now, there have been numerous works for developing accurate short-term load forecasting. Many statistical methods, including linear or multiple regression, autoregressive integrated moving average (ARIMA) models [3, 4], Kalman filtering technology [5], and exponential smoothing models [6, 7], have been applied in this filed, and some good results are obtained. These methods are easy to implement, but the drawback is that they hardly capture the sudden variety among time series and achieve the expected performance because of their linear definition theoretically. Recently, artificial neural network (ANN) models have been extensively explored to receive more higher forecasting accuracy and overcome the above limitations [8–10]. Due to their excellent learning and generalization capabilities, neural networks have been found to be the first-choice candidates in the field of time-series forecasting. For example, hourly short-term electricity load forecast using ANN in England was presented and showed a very good prediction that the mean absolute percentage errors (MAPE) are 1.38% for weekdays and 1.39% for weekends [10]. However, the network training of ANN requires huge amounts of data, and its interpretability of the prediction results is poor [11]. Apart from neural network-based models, there are also many machine learning algorithms producing superior abundant results in some applications, such as atmospheric rainfall forecasting [12], financial forecasting [13], and tourism forecasting [14]. The machine learning algorithms mainly include support vector regression (SVR) [12, 15, 16], decision trees [17–20], random forest (RT) [21], and gradient boosting regression trees (GBRT) [22]. The SVM can solve the practical problems such as small sample, nonlinear, high, and local minimum point, but this method cannot determine the input variables effectively and reasonably, and it has slow convergence speed and poor forecasting results while suffering from strong random fluctuation time series [15]. Therefore, Fan et al. proposed an improved SVR model hybridized with the empirical mode decomposition method and autoregression, which can both provide more accuracy forecasts and interpretability [16]. Recently, the application of decision tree algorithm to load forecasting has received some excellent results. Decision tree is a kind of tree structure used in regression and classification, which is also called regression tree used for regression. It allows to extract if-then rules and clarify the nonlinear relationship between inputs and outputs easily. The most significant favor of regression trees is the ability of easy development and interpretation due to their nonparametric design. Bootstrap aggregated (bagged) is an ensemble method that can solve the overfitting problem for regression trees. It builds multiple trees repeatedly based on resampling with replacement and then integrates these decision trees to vote to obtain better prediction accuracy. In fact, bagged regression trees (BRT) have demonstrated the applicability and effectiveness of load forecasting in some studies. For example, Carmen et al. evaluated the effectiveness of ensemble methods (bagging, random forest, conditional forest, and boosting) based on regression trees in short-term load forecasting [21]. Four ensemble models were applied to the electricity consumption of a campus university in Cartagena, Spain. Accuracy results for four models showed that bagging and random forest provided the best accuracy in the training dataset. Accordingly, the BRT model will be employed in this paper.

It is well-known that the error of short-term load forecasts mainly derives from the special days when the load shape deviates significantly from the normal days, such as weekends, public holidays, and days preceding and following holidays [23–25]. Load observed on normal days is regarded as normal load, whereas the load observed on special days is regarded as anomalous load. Typically, while the load data on special days have been used in the training of prediction models, they have been eliminated from testing set [26]. Darbellay and Slama [27] divided the data into working days and holidays to forecast separately but still did not achieve good results during holidays. It can be seen that the modelling of anomalous load had usually been overlooked in many previous works, and there are some reasons summarized as follows: (1) load shapes on special days are quite different from those of normal days; (2) the lack of observation samples of special days leads to insufficient training; (3) different special days exhibit different load variation patterns [28]. For this, several works are done for developing accurate short-term load forecasting methods for special days. For instance, the new fuzzy regression model was applied, and it improved the prediction accuracy of the holidays falling [29]. Although, forecasting errors of holidays falling on Saturday or Monday were bigger than those of other days, they further studied linear regressive analysis and relative coefficient analysis to make the prediction better. In fact, the actual power load data had shown that the load patterns on the days before and after the holidays were also different from normal days [30]. Considering the load drop due to the proximity of the forecast day, Lamedica et al. applied a special rule with adding a distance variable of a specific day from Sunday or holiday day [31]. On the days before and after public holidays, the accuracy of load forecasting had some improvement. And, Pardo et al. employed dummy variables to capture the weekly periodicity, yearly periodicity, and holiday effects and used additional dummy variables to represent the days following public holidays that often exhibit different load profile from that of normal days [32].

In general, some different special days are usually classified as the same type to avoid overparameterization in the method to deal with anomalous load that involves the use of dummy variables [28, 33]. The classification of special days should rely on the assumption that the load shapes of different special days can be considered similar and would remain similar for many years. In addition, it is extremely necessary to analyze local data in detail and choose a suitable method, because the load variation patterns have large differences in different regions. In our previous work [34], the load time-series measured from 2016 to 2018 in Qingdao was investigated in order to make a prediction more accurately by using an ANN model combined with eight input variables. Among these inputs, for the sake of simplicity, public holidays and weekends were assumed as one group of nonworking days. Then, one dummy variable was generated in the load prediction model, including 0 for nonworking days and 1 for working days. However, there are some differences in load profile during public holidays and weekends.

In this paper, it is aimed to make contributions to address the issues on short-term load forecasting for special days. First, the applicability of BRT model combined with eight predictors, which had been used in [34], is investigated to forecast short-term load in Qingdao. Then, the Chinese national holidays are classified into five different categories to better mining of different load profiles. According to the analysis result of the load data during the holidays from 2016 to 2017, these special days are divided into three types including statutory days, bridging days, and days preceding and following holidays, which are called proximity days. As a result, an indicator variable is proposed to represent different special days. Lastly, the BRT model combined with this proposed indicator variable for special days is tested on load time-series measured from 2018.

The remainder of this paper is structured as follows: Section 2 introduces the BRT forecasting model. Then, a brief description of the load characteristic can be found in Section 3.  Section 4 provides the empirical comparison between BRT model and ANN model. In Section 5, the detailed analysis of the load characteristic for special days is given. At the same time, a new indicator variable is proposed to distinguish different special days. In Section 6, the BRT model combined with this new indicator variable is tested on the load data for whole year and holiday periods in 2018.  Section 7 provides some discussion of the prediction results. Finally, main conclusions are presented in Section 8.

## 2. Bagged Regression Trees

Decision tree was firstly proposed in 1984 by Breiman, which is widely used in both classification problems and regression analysis [35]. When used for classification, each leaf node in the decision tree represents a category, and when used for regression, each leaf node represents a predicted value, which is continuous. Considering the practical application of this study, we only introduce the case of regression.

### 2.1. Regression Tree

**Figure 1:**
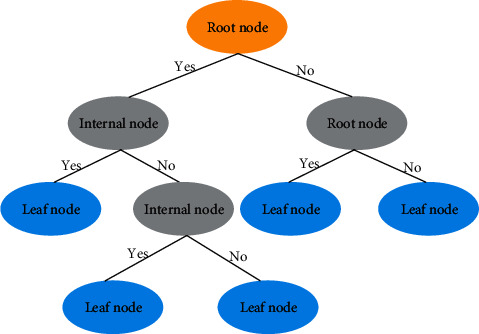
The structure of a simple regression tree.

The regression trees represent a mapping between object attributes and object values. Each node in the tree represents an object, each fork path represents the value of a possible property, and each leaf represents the value of the object represented by the path taken from the root to that leaf. The structure of a simple regression tree is shown in Figure 1. Specifically, each regression tree represents a division of the feature space and the output value on the division unit.

Assume a training data set: *D*={(*x*_1_, *y*_1_), (*x*_2_, *y*_2_),…, (*x*_*i*_, *y*_*i*_)}, *x*_*i*_ ∈ *R*^*n*^, *y* ∈ *R*. The object of the regression problem is to construct a function *f*(*x*), which can fit the elements in the data set *D* to minimize the loss function.

(1) $$\begin{array}{c} \text{object}=\min\left(\frac{1}{n}\text{Loss}\left(f\left(x_{i}\right)-y_{i}\right)\right). \end{array}$$

In this context, the mean square error (MSE), which is common for regression problem, is used as the loss function and shown in the following equation:

(2) $$\begin{array}{c} \text{MSE}=\sum_{i=1}^{n}{\left(f\left(x_{i}\right)-y_{i}\right)}^{2}. \end{array}$$

Suppose that a constructed regression tree has *M* leaves, which means that the tree divides the input space *X* into *M* units *R*_1_, *R*_2_,…, *R*_*M*_, and it also means that there are at most *M* different predictions. The MSE minimization formula of the tree is as follows:

(3) $$\begin{array}{c} \min\frac{1}{n}\sum_{m=1}^{M}\underset{x_{i}\in R_{m}}{\sum }{\left(c_{m}-y_{i}\right)}^{2}, \end{array}$$

where *c*_*m*_ represents the predicted value of the *m*-th leaf:

(4) $$\begin{array}{c} c_{m}=\text{ave}\left(y_{i}|x_{i}\in R_{m}\right). \end{array}$$

to minimize the overall MSE of this regression tree, that is, to minimize the MSE of each leaf.

Therefore, in each division, the splitting variable and splitting point that minimize the sum of MSE of each leaf should be selected. This content adopts the heuristic method to traverse all splitting variables and splitting points and then select the case with the smallest sum of leaf nodes MSE as the division.

Traverse the variable *j*, scan the splitting point *s* for the fixed splitting variable *j*, and select the pair (*j*, *s*) that minimizes the following formula:

(5) $$\begin{array}{c} \min\left[\underset{c_{1}}{\min}\sum_{x_{i}\in R_{1}\left\{j,s\right\}}{\left(y_{i}-c_{1}\right)}^{2}+\underset{c_{2}}{\min}\sum_{x_{i}\in R_{2}\left\{j,s\right\}}{\left(y_{i}-c_{2}\right)}^{2}\right]. \end{array}$$

Use the selected pair (*j*, *s*) to divide the area and determine the corresponding output value:

(6) $$\begin{array}{c} R_{1}\left\{j,s\right\}=\left\{x|x^{\left(j\right)}\leq s\right\}, \end{array}$$

(7) $$\begin{array}{c} {\hat{c}}_{m}=\frac{1}{N_{m}}\sum_{x_{i}\in R_{m}\left(j,s\right)}y_{i},\;x\in R_{m},m=1,2, \end{array}$$

where *N*_*m*_ refers to the number of elements in the *m*-th region. Continue to call formulae (5)–(7) on the two divided subregions until the stop condition is met.

Finally, the input space *X* is divided into *M* regions *R*_1_, *R*_2_,…, *R*_*M*_, and generate a regression tree:

(8) $$\begin{array}{c} f\left(x\right)=\left\{\begin{array}{c} {\hat{c}}_{1},\;x_{i}\in R_{1}\left\{j,s\right\}\\ {\hat{c}}_{2},\;x_{i}\in R_{2}\left\{j,s\right\}\\ {\hat{c}}_{3},\;x_{i}\in R_{3}\left\{j,s\right\}\\ \cdots \\ {\hat{c}}_{M},\;x_{i}\in R_{M}\left\{j,s\right\}. \end{array}\right. \end{array}$$

The regression tree algorithm has the potential to simulate highly nonlinear and complex relationship between the input variables and the outputs. And, it can be considered as a base learner in the field of machine learning. In addition, the constructed regression tree has the ability to accurately extract features from data with large differences in data types. However, regression trees can be extremely nonrobust and generally provide less forecasts accuracy than some of the other regression methods. Fortunately, these disadvantages can be easily improved by aggregating many regression trees using ensemble methods, such as bootstrap aggregating (bagging), random forests, and boosting [35]. The bagging ensemble method is used in this study.

### 2.2. Bagging

The principle of bagging ensemble method, which was primarily designed by Breiman [36], is to construct and combine multiple individual learners to accomplish the final prediction task. This ensemble learning method is often used to reduce the variance of regression trees and remedy the overfitting problem in the single tree. The specific steps can be summarized as follows:

  Step 1: randomly generate *T* new sample sets of the same size as the training sets using bootstrap method.
  Step 2: generate the corresponding regression tree based on the method mentioned above on each training set. For example, 50 trees are used in the bagging trees models in this work.
  Step 3: apply the generated regression trees to the test sample to obtain the predicted values. And the final predicted values can be obtained by averaging the values of each trees. The prediction of the bagging trees model is expressed as

(9) $$\begin{array}{c} \hat{h}\left(x\right)=\frac{1}{T}\sum_{t=1}^{T}{\hat{f}}_{t}\left(x\right), \end{array}$$

where *f*_*t*_ is the predicted value based on the *t*-th tree.

**Figure 2:**
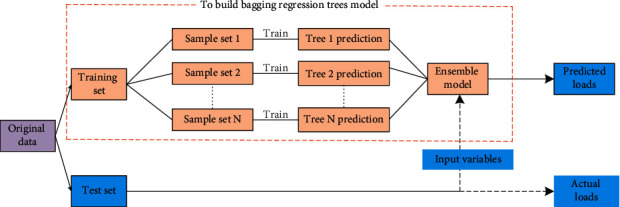
Flowchart of the basic idea of bagged regression trees prediction.

The overall flowchart of the basic idea of bagged regression trees prediction is shown in Figure 2.

As an ensemble algorithm, the bagging algorithm is mainly used to randomly extract mutually independent training sets, and each round of training is parallel to improve the training speed. Theoretically, it can be proved that the variance of prediction can be reduced to 1/*N* (*N* is the number of learners) of the original variance (single learner) [37]. Therefore, prediction variance can be reduced by using multiple learners.

## 3. Load Characteristic

In this paper, three years of hourly load in Qingdao are used from January 1, 2016, to December 31, 2018, which accumulate 26, 304 observation points. The period from January 1, 2016, to December 31, 2017, is employed for estimation purposes (in-sample), and the data observed in 2018 are left for forecast evaluation (out of sample). The complete load profile is shown as follows.

### 3.1. Intrayear Seasonality

**Figure 3:**
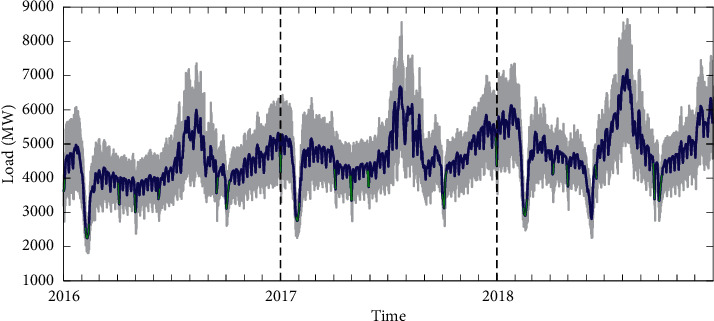
Raw load profile for Qingdao from 1 January 2016 to 31 December 2018. The gray fluctuation line describes the raw hourly load, the blue curve shows the profile of daily average load, and the green curved section represents some public holidays.

It can be seen from Figure 3 that the load exhibits a recurring-year pattern because of the seasonal effects, which is referred to as the intrayear seasonality. The summer load is the highest with the largest fluctuations in the whole year. In contrast, the load levels in spring and autumn are low and stable. However, there are two completely different variation trends in winter: it gradually increases during the transition from autumn to winter and decreases sharply at the end of January and early February. Overall, the annual average load shows an upward trend. In addition, there are intraday and intraweek seasonality in the load sequence, which has been confirmed by the power spectral density in [34]. Moreover, it is noted that the load values during public holidays marked by green curve are considerably lower than those on normal days, and the load values are the lowest during the Chinese Lunar Spring Festival. Specific dates of all public holidays in China can be found in Table 1.

### 3.2. Intraday and Intraweek Seasonality

**Figure 4:**
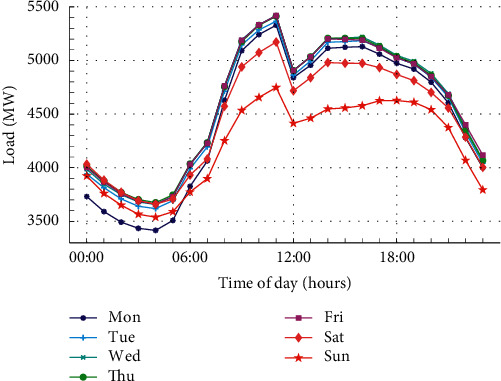
Average intraday profile for each day of the weeks from 2016 to 2017.

The average intraday profile for each day of the week from 2016 to 2017 is shown in Figure 4. It can be seen from the figure that the load values during weekend are lower than those during weekdays, and the load value on Sunday is the lowest. However, the daily change pattern is consistent throughout the week. It is worth noting that the load value at 12 am shows a sharp decline. The existence of this phenomenon is also one of the reasons why statistical models cannot be used to make predictions. This is due to the fact that the statistical approach is based on the theory of polynomials, which cannot capture this sudden change well.

In this paper, the eight predictors are adopted based on the consideration of power spectrum analysis and mutual information [34]. The eight predictors include hour of day, load from the same hour in the previous day, previous day's average load, day of week load from the same hour and same day from the previous week, a dummy variable indicating whether it is a working day or nonworking day, temperature, and humidity.

## 4. Empirical Comparison

### 4.1. Evaluation Criteria

To assess the forecasting performance of the proposed model, four well-known accuracy indexes, including mean average error (MAE), MSE, MAPE, and root mean squared error (RMSE), are used in this study and are shown as follows:

(10) $$\begin{array}{c} \text{MAE}=\frac{1}{N}\overset{N}{\underset{i=1}{\sum }}\left|y_{i}-{\hat{y}}_{i}\right|,\\ \text{MAPE}=\frac{1}{N}\overset{N}{\underset{i=1}{\sum }}\left|\frac{y_{i}-{\hat{y}}_{i}}{y_{i}}\right|,\\ \text{RMSE}=\sqrt{\frac{1}{N}\sum_{i=1}^{N}{\left(y_{i}-{\hat{y}}_{i}\right)}^{2}}, \end{array}$$

where *N* is the total number of forecasting results; *y*_*i*_ is the actual load at point *i*; and ${\hat{y}}_{i}$ is the forecast load at point *i*.

### 4.2. Empirical Comparison

To demonstrate the applicability of the BRT model with eight predictors to forecast short-term load, the empirical comparison between the ANN model used in our previous paper and BRT model is carried out, based on an evaluation of their simulation accuracy for the out of-sample period, which consist of load observations of all hours in 2018. The most classic backpropagation neural network (BPNN) was used in a previous work. Similarly, MAPE is used as the forecasting accuracy index. The predictors considered in the basic experiment (Be) and four comparative experiments (Ce) are the same as before. Note that the only difference between the above two types of experiments is due to the input variables used in the prediction model. So, this will not be repeated here, and more details about ANN model and experiments can be found in [30].

The forecasting accuracy of the Be and Ce is presented in Table 2. Note that the BRT model shows slightly higher accuracy in almost all experiments except for Ce-3. More specifically, the prediction accuracy of the BRT model in five experiments is 0.11% lower than that of the ANN model on average. Particularly, for Ce-4 that considers eight features, the MAPE value of the BRT model reaches 3.15%, which is 0.3% lower than that of the ANN model. These results illustrate not only the effectiveness of the BRT model for load forecasts, but also the adaption of the above eight predictors to the BRT model. However, there still exists a slightly larger error on some special days. Therefore, a targeted study on the load variation pattern on special days is conducted below.

## 5. Load Characteristic for Special Days

The load profiles of public holidays were significantly different from those of normal days, and different public holidays have different load profiles, so it is necessary to analyze the load data of each holiday separately. We identify a total of three categories of seven public holidays in China via the legal public holidays' arrangement and the actual situation. The current standards for some public holidays are one day, such as New Year Day, Qingming Festival, May Day, Dragon Boat Festival, and Mid-Autumn Festival, but it is usually extended to three days in the form of an adjustment or continuous holiday. We refer to these holidays as basic public holidays and classify them as category *A*. In the same way, Chinese National Day holiday, which originally stipulated a three-day holiday, is usually extended to one week, and we classify it as category *B*. As mentioned above, the load variation during the Chinese Lunar New Year is indeed remarkably different from other public holidays. At the same time, the Chinese Lunar New Year with longer holiday period is classified as Category *C*. Although the New Year's legal holiday is the same as the National Day, it is generally extended to one to two weeks or even longer.

### 5.1. Category A: Basic Public Holiday

The basic holidays in 2016 and 2017 are classified into three different types, because the statutory day of the basic holiday may occur on any day during the holiday periods.

#### 5.1.1. Statutory Day Occurred on the Third Day of the Holiday Period

**Figure 5:**
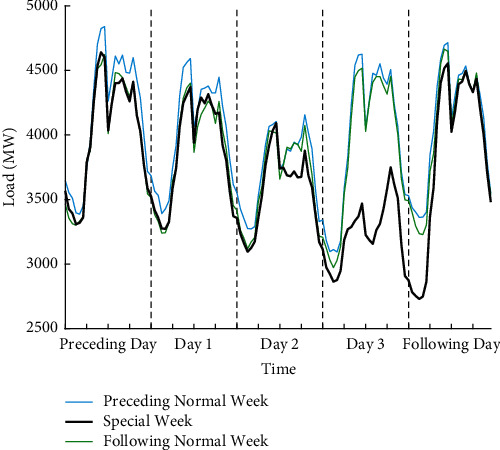
Load profile for five days of special week including the Qingming Festival (April 2–4, 2016), five normal days (March 25–29, 2016) from the preceding week, and five normal days (April 8–12, 2016) from the following week. The blue line represents the hourly load on normal week preceding holiday, the black line represents the hourly load on special week, and the green line represents the hourly load on the normal week following holiday.

Using the Qingming Festival in 2016 as a representative case, its statutory holiday is one day, but as shown in Figure 5, it is extended to three days for practical reasons. It can be seen from the figure that the load on statutory day (April 4, Monday, Day 3) is noticeably lower than that of the other two days (April 2–3, Day 1 and Day 2) during the holiday, namely, bridging days. Specially, there are huge differences for load between the statutory day and the normal Monday, which are working days. It is worth noting that load values during the two bridging days and load values during weekends from the normal weeks are very similar. Moreover, the days both preceding and following holiday (April 1 and April 5), which are referred to as proximity days in this study, exhibit similar load variation patterns with normal days. Therefore, proximity days can be treated as normal days in modelling.

#### 5.1.2. Statutory Day Occurred on the Second Day of the Holiday Period

**Figure 6:**
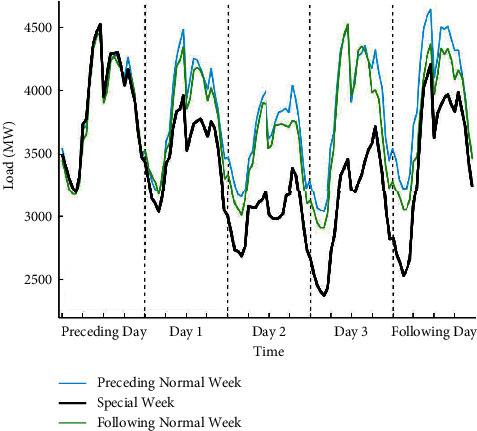
Load profile for five days of special week including the May Day (April 30–May 2, 2016), five normal days (April 22–26, 2016) from the preceding week, and five normal days (May 6–10, 2016) from the following week. The line representation is the same as in Figure 4.

In Figure 6, the load profile for special days is displayed, including the May Day holiday and bridging days, five normal days from the preceding week, and five normal days from the preceding week. As excepted, the statutory day (May 1, Sunday, Day 2) has the lowest load value, followed by the bridging days (April 30 and May 2, Day 1 and Day 3). There are some differences between load values in the bridging days and the days from normal weeks. But the load pattern of bridging days is very similar with normal weekends. Moreover, proximity days and the corresponding days from normal weeks are observed to have particularly similar intraday load pattern, which is generally consistent with the above analysis.

#### 5.1.3. Statutory Day Occurred on the First Day of the Holiday Period

**Figure 7:**
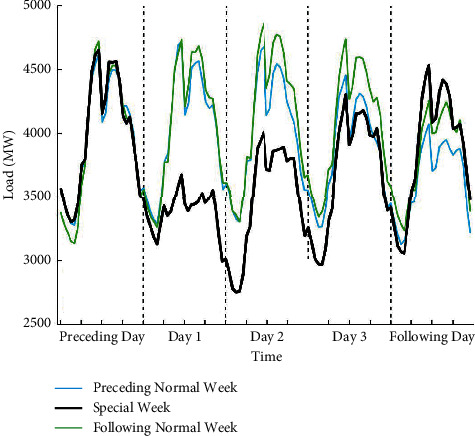
Load profile for five days of special week including the Dragon Boat Festival (June 9–11, 2016), five normal days (June 1–5, 2016) from the preceding week, and five normal days (June 15–19, 2016) from the following week. The line representation is the same as in Figure 4.

As shown in Figure 7, due to the impact of the Dragon Boat Festival, the load values during the entire holiday period have dropped significantly. The load values on statutory days (June 9, Day 1) are much lower than those on bridging days (June 10-11, Day 2-3). The two bridging days are Friday and Saturday; and the load pattern is similar to that of the normal weekend (June 4-5 or June 18-19). As a result, the bridging days can be considered the same as normal weekends here.

**Figure 8:**
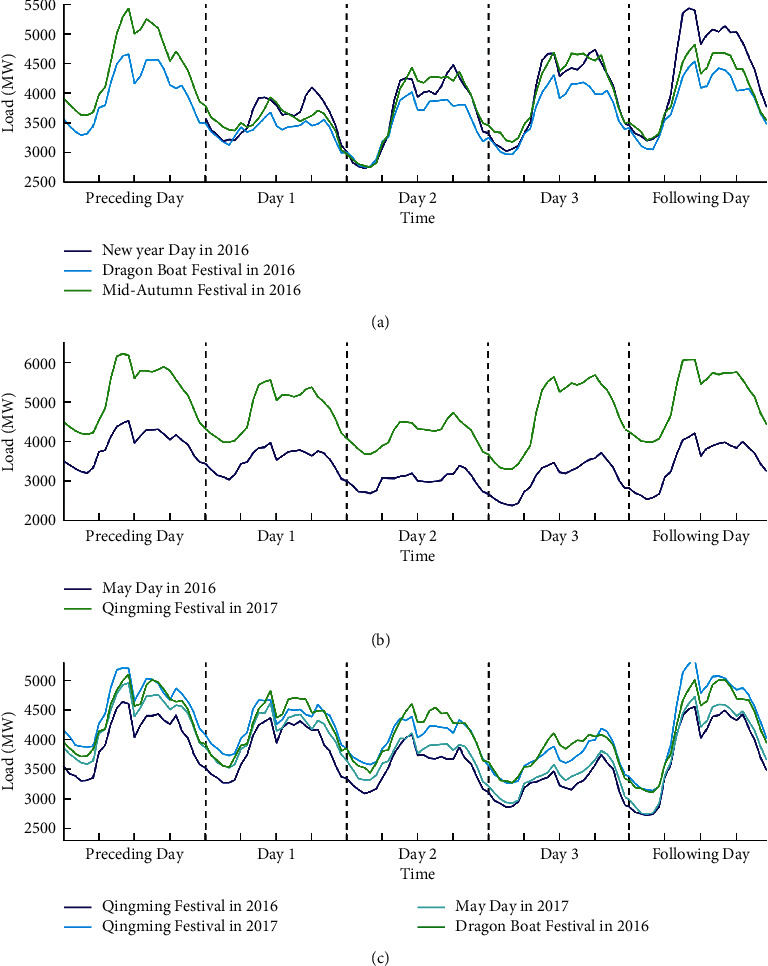
Three types of basic holidays in 2016 and 2017. (a–c) The load profile on basic holidays that statuary day occurred on the first day, second day, and third day, respectively.

The load profiles during three different type of basic holidays from 2016 to 2017 are presented in Figure 8. As excepted, the lowest peak of load values during each holiday period appears on statutory day, which should be considered as abnormal variation. Moreover, the load pattern on bridging days can be roughly regarded as the same as normal weekend. In addition, load pattern on proximity day of the basic holiday period is similar to that on normal days, which does not have special processing in modelling. Therefore, one indicator variable is proposed to capture the above abnormal patterns, including 2 for statuary days, 1 for proximity and normal working days, and 0 for both bridging days and weekends.

### 5.2. Category B: Chinese National Day

**Figure 9:**
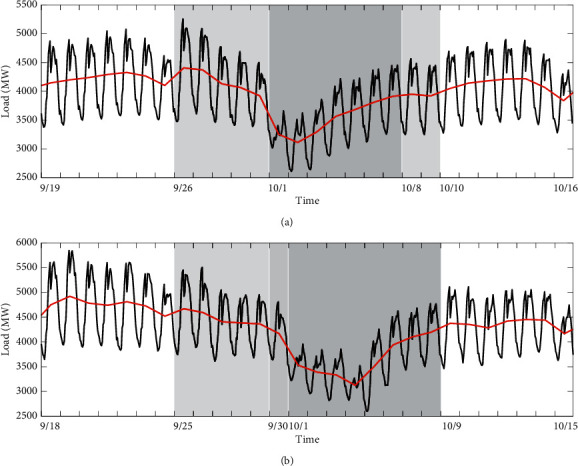
(a) Load profiles for 4 weeks including Chinese National Day Holiday (October 1–7), two weeks in which the load is affected (September 26–30 and October 1–7), and two normal weeks (September 19–25 and October 10–16), observed in 2016. (b) Load profiles for 4 weeks including Chinese National Day Holiday (October 1–8), a working day (September 29), two weeks affected (September 25–29), and two normal weeks, observed in 2017. The black curves represent the hourly load, and the red curves represent daily average load.

As a relatively important holiday, the statutory holiday of Chinese National Day lasts for three days, but it is usually extended to one week. As shown in Figure 9(a), the load values on the whole holiday period (October 1–7) in 2016 are significantly lower than those on the other days, especially on statutory days (October 1–3). Moreover, the load values on the five days preceding holiday are observed to show a downward trend, which is undoubtedly under the influence of the holiday period. Fortunately, the normal intraweek variation pattern can be found before September 25 and after October 9. It should be noted that October 8 and 9 has changed from weekends to working days to compensate for the loss of working days due to public holidays. Thus, the load level and variation on these two days are slightly different from those of normal weekends, such as September 24 and September 25. In 2017, the overall load variation during the holiday period is almost the same as in 2016, but as shown in Figure 8(b), the load values drop on a special day in 2017 (i.e., September 30, 2017, the Mid-Autumn Festival), which should be considered as an inevitable special situation. From what has been discussed above, this public holiday has affected the load pattern for two weeks.

### 5.3. Category C: Chinese Lunar Spring Festival

**Figure 10:**
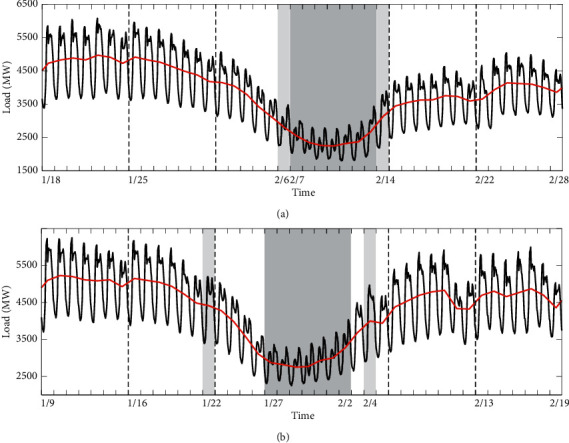
Load profiles for Chinese Lunar Spring Festival in 2016 and 2017. The red curves represent daily average load. The dark gray shaded parts represent holiday, and the light gray shaded parts represent working days. The line representation is the same as in Figure 8.

It can be clearly seen from Figure 10(a) that the load values during the holiday period (February 7–13) are lowest among the four weeks, especially on statutory days (February 7–9). The load values for almost two weeks preceding the holiday show a downward trend, and the third week preceding the holiday returns to normal.

Based on the above visual inspection of the load data on two long holidays, we notice that the load on statutory days is lowest on whole holiday, and the load on the bridging days (other four days of holiday except for statutory days) tends to be lower than that of the normal days, but higher than that of statutory days. In addition, load variation on some proximity days including the one-week preceding holiday is also abnormal. Therefore, these special days are marked by an indicator variable, including 2 for statutory days, 1 for normal working days, and 0 for bridging days, proximity days, and weekends. The method of incorporating subjective judgment in forecasting models using some specific rules has been widely employed in load forecasting field [28].

## 6. Experiments

In this section, the comparison experiments and experimental results are presented for prediction hourly load for 2018 in Qingdao. All experiments are executed under the MATLAB environment on a PC platform, with 2 Intel Core dual core CPUs (2.4 GHz) and 8 GB RAM in Windows 10 operating system.

### 6.1. Comparison Experiments

The comparison experiments between the BRT-1 model and the BRT-2 model are carried out, which is used to demonstrate the effectiveness of the proposed indicator variable explained in Section 5 for improving prediction accuracy, especially on special days. The BRT-1 model refers to the BRT model considering eight features used in Ce-4 (see Section 3). For BRT-2 model, the dummy variable used in BRT-1 model is replaced with the new indicator variable that can capture the abnormal variation of load values on special days. The specific model inputs of BRT-1 and BRT-2 are shown in Table 3.

### 6.2. Experimental Results and Analysis

#### 6.2.1. Results for All Hours in 2018

As shown in Table 4, the MAE, MSE, MAPE, and RMSE are adopted as the forecasting accuracy indexes of model. It can be seen from this table that the BRT-2 model is superior to BRT-1 model in terms of MAE, MSE, and RMSE. These results fully justify and highlight the importance of incorporating the proposed indicator variable for special days in the modelling for the load data of Qingdao. As the forecast results in terms of MAPE have not been improved overall, the MAPE of hourly load using the BRT-1 and BRT-2 model is shown in Figure 10 in a more specific form.

**Figure 11:**
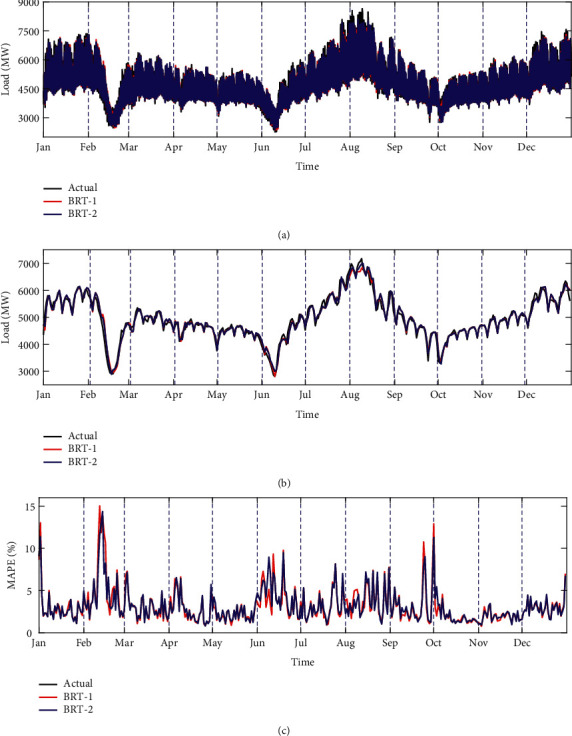
(a) Comparison for hourly load between actual and forecast loads; (b) comparison for average hourly load of each day between actual and forecast loads; (c) MAPE across hourly load using the BRT-1 and BRT-2 model.

In Figure 11(a), there is no significant difference between the forecast and the actual load values using the BRT-1 and BRT-2 model. But, we can observe from Figure 11(b) that BRT-2 model shows better prediction results in August than the BRT-1 model. For further comparison, Figure 11(c) presents the forecasting accuracy of the BRT-1 and BRT-2 model. Obviously, the BRT-2 model achieves smaller MAPE values on some days, such as the few days in January, February, June, August, September, and October. Encouragingly, the BRT-2 model is considerably more accurate on special days, including the Chinese national day holiday and some proximity days, than the BRT-1 model. Therefore, in terms of the above four accuracy indexes, the prediction results of the BRT-2 model have improved to varying degrees.

#### 6.2.2. Results for Basic Holidays

**Figure 12:**
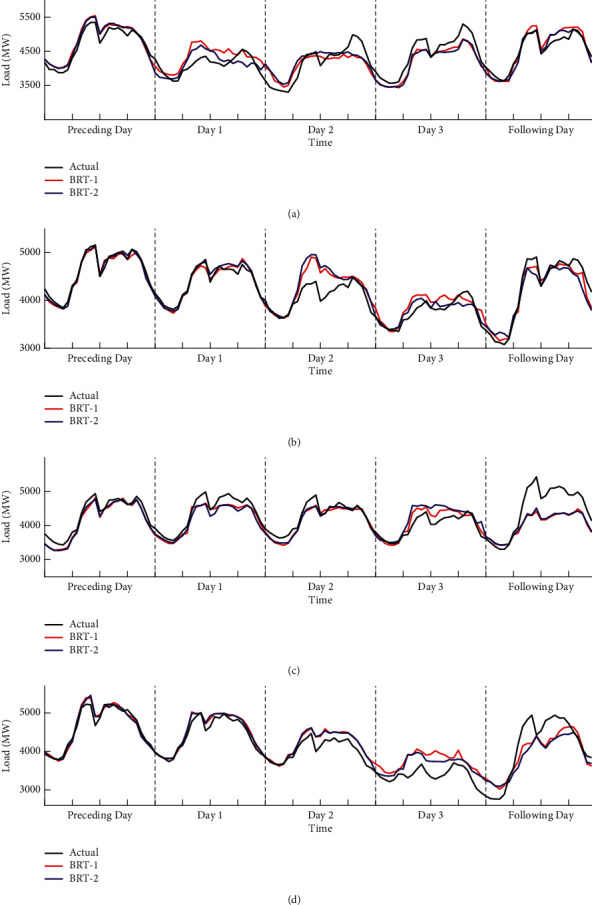
Comparison for basic holidays including (a) Qingming Festival, (b) May Day, (c) Dragon Boat Festival, and (d) Mid-Autumn Festival between actual and forecast loads.

The comparison of four basic holidays between actual and forecast loads is presented in Figure 12. As expected, the BRT-2 model fits better on three statutory days of Qingming Festival, May Day, and Mid-Autumn Festival than on the statutory day of the Dragon Boat Festival holiday period. The reason that the predicted load values are higher than the actual load values in 2018 may be due to the fact that the load pattern of these holidays in 2016 and 2017 is not the same as that in 2018, which is also a normal phenomenon. Overall, the BRT-2 model can better capture the abnormal variation of the statutory days during the basic holiday, when compared with the BRT-1 model. In addition, there are slight improvements in some proximity days using the BRT-2 model.

#### 6.2.3. Results for Chinese Lunar Spring Festival

**Figure 13:**
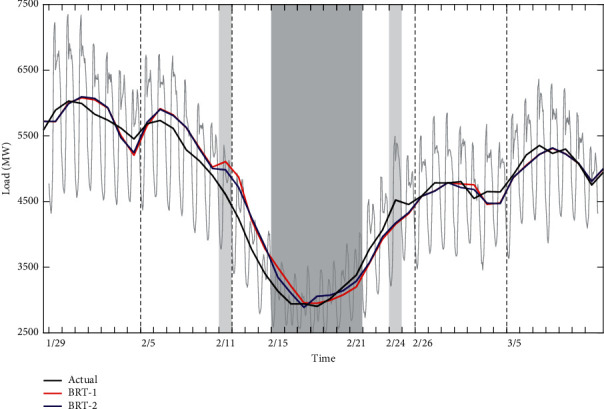
Comparison for Chinese Lunar Spring Festival between actual and forecast loads in 2018. The black curves represent the actual load, the red curves represent the predicted load of BRT-1 model, and the blue curves represent the predicted load of BRT-2 model.

Figure 13 shows the comparison between actual and forecast loads using BRT-1 and BRT-2 model during Chinese Lunar Spring Festival. Encouragingly, both prediction models capture the overall situation of holiday load values well. Specifically, load values predicted by BRT-2 model showed a slightly upward trend on February 11, which is a working day. However, the BRT-2 model fails to capture the load variation on February 24, which is also a working day. And, the responses of the two models lag behind the normal load by about one day. Since the model has learned and trained the load data features of 2016 and 2017, some forecasting errors in 2018 are normal and in line with reality. However, there may be special characteristics in 2018 that are different from the previous two years.

#### 6.2.4. Results for Chinese National Day

**Figure 14:**
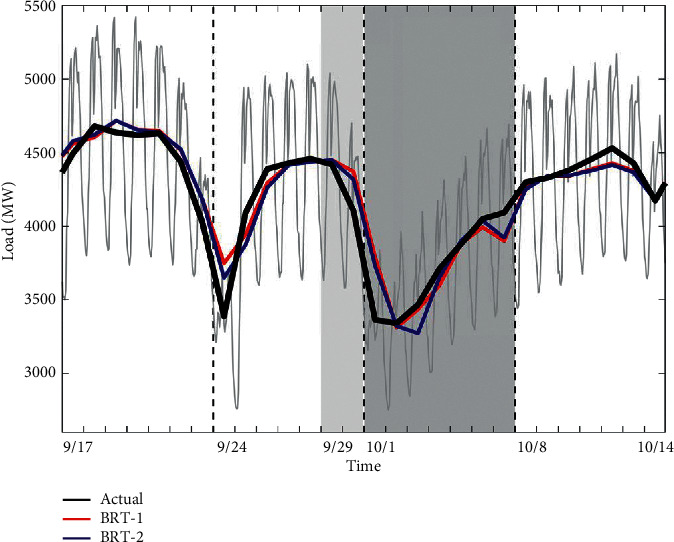
Comparison for Chinese National Day between actual and forecast loads in 2018. The line representation is the same as in Figure 12.

The forecast load values for the four weeks around the Chinese National Day using BRT-1 and BRT-2 model are presented in Figure 14. As shown in Figure 14, the BRT-2 model roughly captures the pattern of changes throughout the holiday and has a slight advantage over the forecasting error of the BRT-1. The most significant improvement of the BRT-2 model can be found in September 24, which is a proximity day. This is because the BRT-1 model makes no attempt to model the proximity days. However, the response of the BRT-2 model also shows a lagging trend from September 29 to October 3.

## 7. Discussion

Overall, compared to the BRT-1 model with the original dummy variable, the BRT-2 model with the proposed indicator variable produced the more accurate predictions on special days. For these days, the MSE obtained with BRT-2 is 4.8% lower than the MSE obtained with BRT-1, which makes no attempt to model bridging days and proximity days. However, what needs special explanation is that although the forecast results on few days are not as expected, the overall situation is still relatively good. Since the model has learned and trained the load data features of 2016 and 2017, there may be differences between the forecasting results in 2018 and those in 2016 and 2017. The slight discrepancy between model learning and reality is also in line with the actual situation. In addition, the lag in the response of the prediction model appears during the two long holiday periods of the Chinese New Year and the National Day. The reason for this phenomenon may be due to the high randomness of the load sequence during the two long holiday periods and the few available test data. In further work, it is possible to make reasonable adjustments or further improvement on the model based on actual applications.

## 8. Conclusion

The accuracy of short-term load forecasting on special days is further improved by incorporating the proposed indicator variable in the BRT model. Thus, the conclusions of this paper can be stated as follows: (1) under the same model inputs and test data, the BRT model is slightly outperforming for load forecasting work than the ANN model. (2) A significant decline in load values happens on the statutory days during any public holiday, followed by that on bridging days. Moreover, load values on some proximity days also exhibit different load pattern from normal days. A new indicator variable is proposed to capture the abnormal variations. (3) The BRT model with the proposed indicator variable performs better than the BRT model with the original dummy variable, which indicates that the proposed indicator variable is extremely effective for the prediction accuracy of special days.

Based on the above research, the prediction results of special days have been fundamentally improved, and some work has been done in the past for the selection of predictors. In future work, our research focus will shift to forecasting models. Considering the predictors used in this study, some improved algorithms will be adopted and compared for short-term load forecasting, such as other boosted regression tree [38], SVR with empirical mode decomposition and autoregression [16], SVR with chaotic GASA algorithm [39], LSSVM, LSSVM with fuzzy time series, and global harmony search algorithm [15].

## Figures and Tables

**Table 1:** Specific dates of public holidays in China.

Public holidays	2016	2017	2018
New Year's day	1/1–1/3	2016/12/31–2017/1/2	2017/12/30–2018/1/1
	**Fri** Sat Sun	Sat **Sun** Mon	Sat Sun **Mon**

Chinese Lunar Spring Festival	2/7–2/13	1/27–2/2	2/15–2/21
	Sun **Mon** Tue	Fri **Sat** Sun	Thu **Fri** Sat

Qingming Festival	4/2–4/4	4/2–4/4	4/5–4/7
	Sat Sun **Mon**	Sun Mon **Tue**	**Thu** Fri Sat

May Day	4/30–5/2	4/29–5/1	4/29–5/1
	Sat **Sun** Mon	Sat Sun **Mon**	Sun Mon **Tue**

Dragon Boat Festival	6/9–6/11	5/28–5/30	6/16–6/18
	**Thu** Fri Sat	Sun **Mon** Tue	Sat Sun **Mon**

Mid-Autumn Festival	9.15–9.17	10/1–10/8 **Sun** Mon Tue	9/22–9/24
	**Thu** Fri Sat		Sat Sun **Mon**
Chinese National Day	10/1–10/7		10/1–10/7
	**Sat** Sun Mon		**Mon** Tue Wed

Note: the statutory days are denoted in bold.

**Table 2:** MAPE of five experiments (%).

Experiment	Be	Ce-1	Ce-2	Ce-3	Ce-4
ANN	3.73	3.66	3.51	3.61	3.45
BRT	3.71	3.54	3.39	3.62	**3.15**

**Table 3:** Model inputs of BRT-1 and BRT-2.

Experiments	Model inputs
BRT-1 (BRT)	*H*(*t*), *L*(*t* − 24), AveL(*t* − 24), Day(*t*), *L*(*t* − 144), DuVar^a^(*t*), Tem(*t*), Hum(*t*)
BRT-2	*H*(*t*), *L*(*t* − 24), AveL(*t* − 24), Day(*t*), *L*(*t* − 144), InVar^b^(*t*), Tem(*t*), Hum(*t*)

^a^The value of DuVar is 0 or 1. ^b^The value of InVar is 0, 1, or 2. Refer to Section 5 for specific settings.

**Table 4:** Summary of results of the forecasting models.

Model	MAE	MSE	MAPE (%)	RMSE
BRT-1 (BRT)	155.86	4.95 × 10^4^	3.15	222.42
BRT-2	154.90	4.71 × 10^4^	3.15	217.07

## Data Availability

The data used to support the findings of this study are included within the article.
